# Oxidative Stress and Down Syndrome: A Systematic Review

**DOI:** 10.3390/antiox14070816

**Published:** 2025-07-02

**Authors:** Goran Slivšek, Sandra Mijač, Ivan Dolanc, Marija Fabijanec, Silvija Petković, Renato Mautner, Karmen Lončarek, Josip Kranjčić, Alenka Boban Blagaić, Marin Marinović, Ksenija Vitale, Donatella Verbanac, Miran Čoklo, Jadranka Vraneković

**Affiliations:** 1 Institute for Anthropological Research, Gajeva ulica 32, 10000 Zagreb, Croatia; ivan.dolanc@inantro.hr; 2 Faculty of Medicine, University of Zagreb, Šalata 3, 10000 Zagreb, Croatia; sandra.mijac@vip.hr (S.M.); renmautner@gmail.com (R.M.); abblagaic@mef.hr (A.B.B.); ksenija.vitale@snz.hr (K.V.); 3 Faculty of Medicine, University of Rijeka, Ulica Braće Branchetta 20, 51000 Rijeka, Croatia; silvija.petkovic@skole.hr (S.P.); karmen.loncarek@uniri.hr (K.L.); marin.marinovic2@gmail.com (M.M.); jadranka.vranekovic@uniri.hr (J.V.); 4 Faculty of Pharmacy and Biochemistry, University of Zagreb, Ulica Ante Kovačića 1, 10000 Zagreb, Croatia; marija.fabijanec@pharma.unizg.hr (M.F.); donatella.verbanac@pharma.unizg.hr (D.V.); 5 School of Dental Medicine, University of Zagreb, Gundulićeva 5, 10000 Zagreb, Croatia; kranjcic@sfzg.hr

**Keywords:** aneuploidy, antioxidants, biomarkers, chromosome disorders, Down syndrome, free radicals, oxidants, oxidative stress, systematic review

## Abstract

Down syndrome (DS), the most common human aneuploidy, is associated with oxidative stress, which contributes to morphological abnormalities, immune dysfunction, cognitive impairment and accelerated ageing. This article aims to provide an overview of the studies on oxidative stress in DS, in particular the investigation of endogenous and exogenous antioxidants, with a focus on endogenous systems. A literature search in MEDLINE and Scopus based on the PRISMA 2020 criteria revealed 41 relevant studies that mainly analysed blood samples (plasma or serum) and occasionally saliva or urine. The findings suggest that oxidative stress in DS is multifactorial and results from an imbalance of superoxide dismutase activity, overexpression of genes on chromosome 21, mitochondrial dysfunction and inflammation. Despite extensive studies over the decades, new sources and mechanisms for oxidative stress in DS continue to emerge, further highlighting the complexity of DS. The recognition that oxidative stress is a hallmark of DS emphasises the need to develop more sensitive and specific methods to detect it and to investigate the associated metabolic pathways in DS in more detail. The expansion of in vivo studies could facilitate the development of targeted interventions aimed at mitigating oxidative damage and ultimately improving outcomes for individuals with DS.

## 1. Introduction

Down syndrome (DS) or trisomy 21 (Ts21) is the most common aneuploidy in the human population and is characterised by a complex and distinct clinical phenotype with intellectual disability, hypotonia and craniofacial dysmorphia. Overexpression of a gene in a region on chromosome 21 leads to an imbalance in the neurological, immunological, endocrine and biochemical processes of the cell. To varying degrees, individuals with DS also exhibit different phenotypes, including disorders of the immune system, respiratory system, endocrine system, gastrointestinal tract, urinary tract and musculoskeletal system. Individuals with DS are prone to premature ageing and earlier development of Alzheimer’s disease [1,2,3,4]. In addition to these diseases, individuals with DS can also suffer from congenital heart defects (CHDs) and have an increased susceptibility to autoimmune diseases, epilepsy, thyroid disease and leukaemia [2,5,6].

The oxidative stress hypothesis offers a possible explanation for several diseases associated with DS. Oxidative stress is a metabolic state of the body caused by an imbalance in the production of free radicals and their reactive metabolites [7,8]. Free radicals are reactive oxygen and nitrogen species that constantly circulate in the body and occur as a side effect of many reactions in the body [9,10]. Studies suggest that free radicals play an important role in the body’s immune response against infectious agents. For instance, the immune system utilises free radicals to fight pathogens. Free radicals have been recognised as important signalling molecules, first for nitric oxide (NO) and then for other reactive species [11]. Furthermore, studies have shown that hormones such as insulin regulate free radical levels and that they can act as important regulators of metabolic processes in the body. It is therefore now clear that free radicals can play a crucial role in various biological processes, including cell signalling, defence against pathogens and the regulation of metabolic pathways [12,13]. However, due to their high reactivity, free radicals can participate in chain reactions in which a single triggering event damages many molecules [14,15]. Under normal conditions, they are removed from the body by antioxidant processes. When these natural mechanisms are disrupted, the radicals accumulate excessively and lead to disease. Mitochondria play an important role in protecting the body from oxidative stress, and their integrity is key to various cellular processes [16,17,18,19]. Reactive oxygen species (ROS) are formed as a by-product of normal cell metabolism during the oxidative reaction of the mitochondrial respiratory chain [20]. It has been shown that this group of molecules could prove an important role in human cellular signalling and defence mechanisms. In moderate amounts, reactive oxygen species play a key role in biological processes such as the destruction of pathogenic organisms, the promotion of wound healing and the repair of damaged tissue [21,22]. Mitochondrial ROS are a significant source of reactive oxygen species trapped in the double membranes of mitochondria. However, both intracellular disorders and external (environmental) factors can lead to excessive production of ROS (superoxide anion (O_2_^•−^), hydroxyl radical (·OH), hydrogen peroxide (H_2_O_2_)). For example, increased oxygen availability, a more intensive metabolism and prolonged stress conditions stimulate ROS production, while increased permeability of the mitochondrial membrane enables the direct release of ROS into the cytosol [22,23]. Mitochondria are particularly susceptible to oxidative damage, as the electrons separated from the electron transport chain in the inner membrane react with oxygen to form the superoxide anion. This anion is unstable and cannot diffuse through the membrane, but it quickly transforms into membrane-permeable H_2_O_2_ [24]. It is also important to emphasise the interaction between ROS and NO, a reactive nitrogen species (RNS). NO can react with O_2_^•−^ to form peroxynitrite (ONOO^−^), a powerful oxidising agent that plays an important role in both cellular signalling and oxidative damage. This interaction between RNS and ROS is crucial for the regulation of numerous cellular processes, including neuronal function and cellular homeostasis. Any disruption of their balance can lead to increased oxidative stress and cellular damage [25,26].

Excessive production of ROS leads to modifications of important biomolecules such as lipids (peroxidation), proteins (aggregation, denaturation), carbohydrates and nucleic acids (changes in the structure of DNA (deoxyribonucleic acid) and RNA (ribonucleic acid)) [22,27,28]. These changes put the body in a state of oxidative stress, which leads to damage and/or changes in cell function and ultimately puts the body in a state of morphological abnormalities, immune dysfunction, cognitive impairment, premature ageing and an increased risk of cancer [1,7,22,29,30]. To prevent damage caused by the excessive production of ROS, cells use an antioxidant defence system, i.e., they use substances that can delay or prevent the oxidation of the oxidisable substrate [31,32]. Antioxidant systems regulate gene expression and signalling pathway connectivity to maintain redox balance and the integrity of cellular components (including lipids, proteins and nucleic acids) [22]. The human body produces several enzymatic and non-enzymatic antioxidants (endogenous antioxidants) that serve to balance the effects of excessive amounts of oxidants [33], and the mechanisms of their action consist of capturing and reduce reactive oxygen species and to repair or replace damaged target molecules [34]. If there is a deficiency of endogenous antioxidants, it is possible to ingest substances that act as a defence mechanism against oxidative stress (exogenous antioxidants), e.g., vitamin C, vitamin E, natural flavonoids, carotenoids and various other compounds, via food and dietary supplements [7,32,35]. Endogenous antioxidants have several key roles in protecting the body: they participate in the neutralisation of excessive amounts of ROS, maintain redox potential homeostasis, participate in the elimination of harmful substances, support the cells of the immune system and contribute to increasing the cells’ resistance to external oxidative factors [32,36,37]. The general endogenous system includes enzymatic antioxidants such as superoxide dismutase (SOD), catalase (CAT), glutathione peroxidases (GPxs) and thioredoxin (Trx), hydrophilic antioxidants such as urate, ascorbate, glutathione and flavonoids, and lipophilic free-radical antioxidants such as tocopherols, carotenoids and ubiquinol [33,38,39]. To assess the relationship between oxidative stress and various diseases and health conditions, including cardiovascular diseases, neurodegenerative disorders and cancer, stable biomarkers, i.e., measurable substances that indicate the presence of oxidative stress in the body, are used [40]. Examples of oxidative stress biomarkers include protein carbonyls (PCs) and advanced glycation end products (AGEs), oxidised low-density lipoproteins, oxidised metabolites, antioxidant enzymes and genes or proteins that are activated in response to oxidative stress [40,41]. In clinical practise, biomarkers are most commonly measured from venous blood and urine samples [41], and somewhat less frequently studies are conducted on saliva, tissue or spinal cord samples [41,42,43]. When measuring biomarkers, different methods are used depending on the type of biomarker and the sample being analysed.

In DS, the presence of an extra copy of chromosome 21 leads to overexpression of several genes that regulate oxidative stress, in particular the enzyme Cu/Zn superoxide dismutase (SOD1; OMIM: 147450) and the amyloid precursor protein (APP) [44,45,46,47]. This overexpression of SOD1 increases the conversion of O_2_^•−^ to H_2_O_2_ without a corresponding upregulation of downstream antioxidant enzymes, including CAT, which directly scavenges H_2_O_2_. This enzymatic imbalance disrupts redox homeostasis and leads to a chronic state of oxidative stress in cells, especially in neurons. Such persistent oxidative stress is a major contributor to the neuropathological and systemic phenotypes that occur in DS. In the central nervous system, oxidative damage to lipids, proteins and nucleic acids impedes neurodevelopment, disrupts synaptic plasticity and accelerates neuronal ageing, leading to cognitive deficits and intellectual disability [48,49,50]. Furthermore, oxidative stress is a key factor in the development of Alzheimer’s disease-like neuropathology by promoting the aggregation of amyloid-beta and hyperphosphorylation of tau, both of which are characteristic of early-onset neurodegeneration in DS. Outside the central nervous system, oxidative stress impairs the functionality of immune cells and can disrupt normal embryonic development, leading to immune dysregulation. In addition, ROS are associated with damage to vascular endothelial cells, possibly contributing to CHDs and metabolic disorders associated with DS [19,51,52]. Studies have shown that individuals with Ts21 have altered mitochondrial structures and reduced mitochondrial connectivity within the mitochondrial network and that mitochondrial dysfunction or damage due to oxidative stress may contribute to the pathogenesis of DS [45,53,54,55].

The aim of this systematic review is to provide an overview of studies conducted in individuals with DS, analysing endogenous and exogenous antioxidants from different types of samples that can serve as biomarkers for the presence of oxidative stress in the body, with a focus on the analysis of endogenous antioxidants in the body of individuals with DS.

## 2. Materials and Methods

This systematic review is based on the updated guidelines for reporting in systematic reviews published in the Preferred Reporting Items for Systematic Reviews and Meta-analyses 2020 (PRISMA 2020) Guidelines [56]. To identify all relevant articles published up to 2024, two leading databases were searched: Medline and Scopus. The articles were found with the following keywords and their combinations: Down syndrome, trisomy 21, oxidative stress, reactive oxygen species and endogenous antioxidants. Once duplicates had been removed, the titles of all remaining articles were thoroughly checked. It was determined what specific information was sought in all included studies. This included the type of study, confirmation that the study was directly related to DS, the inclusion of live human subjects, the presence of a control group, indicators of oxidative stress and specific biomarkers. To extract relevant data from the selected studies, a customised data extraction form was used, which was developed independently and in accordance with the recommendations of the PRISMA 2020 guidelines. This form contained essential information that served as the core criteria for the evaluation of all studies. After a thorough review of available studies, articles analysing endogenous antioxidants as indicators of oxidative stress in individuals with DS, exogenous antioxidants and relevant biomarkers were included in this systematic review. For demonstration in this systematic review, English-language articles were selected that present studies in living subjects (foetuses, children and adults), including study (DS) and control (non-DS) groups with comparable characteristics (age) and with results that can be appropriately read and compared with other relevant articles. Non-English language articles, articles with studies conducted in DS mouse models, articles with post-mortem studies, articles focusing on patient treatment, author manuscripts, randomised controlled trials, case reports, clinical trials, congress abstracts, letters, replies, commentaries, editorials, other systematic reviews and reports with incomplete or unavailable data were removed from the systematic review. The article extraction was carried out by two authors independently on the basis of the inclusion criteria, and any differences were resolved after detailed discussion and comparison, either in dialogue or with the involvement of a third author [57]. To effectively detect and mitigate reporting bias, this systematic review was conducted according to a prospectively developed and publicly available protocol that was registered with PROSPERO (registration number CRD420251012690) and complied with PRISMA 2020 guidelines prior to the start of screening [56,58]. Two independent reviewers systematically assessed the risk of bias in predefined domains for each eligible study and categorised each domain as low, high or unclear risk of bias, after which an overall judgement was made accordingly. Disagreements were resolved by consensus discussions or, if necessary, by consulting a third reviewer. To ensure adherence to this methodology, only studies categorised as low or certain risk of bias were considered for further screening [59,60]. As a result of this transparent and protocol-driven approach, a total of 41 articles were included in this systematic review.

This systematic review has several limitations. The results of the articles analysed were based on a selected sample of subjects, which may pose a methodological problem in some cases (e.g., small number of subjects, variations in the number, sex and age of subjects, etc.). Due to different approaches in the presentation of the numerical values of the analysed parameters in the literature and sometimes the absence of these values in individual articles, these data were also not included in the final summary table. However, the present article was prepared in accordance with the updated PRISMA 2020 guidelines [56] and provides a systematic review of the studies on biomarkers of oxidative stress in individuals with DS.

## 3. Results and Discussion

**Figure 1:**
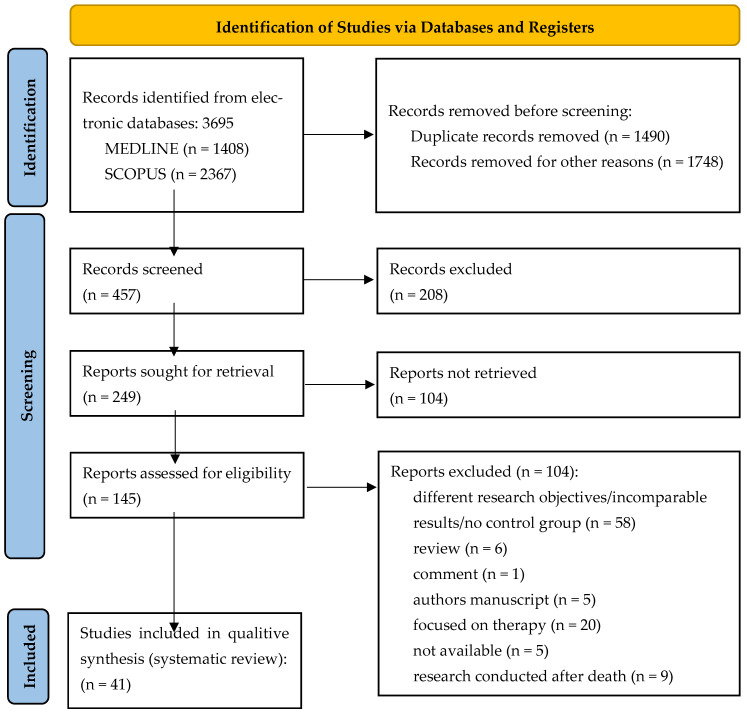
PRISMA 2020 flow chart for new systematic reviews that included searches of databases and registers only.

Initially, a total of 3695 documents were identified in this literature search, of which 1408 were from MEDLINE and 2367 from Scopus. After eliminating 1490 duplicate articles and 1748 articles for other reasons (incomparable results, poorly presented results, samples without a control group, incomplete articles with missing sections, and articles with titles and introductions in English while the rest of the text was written in another language), 457 articles were screened. Additionally, five records were categorised as not available (not available for download or not published). The inclusion and exclusion criteria were then reviewed and finally 41 articles were included in this study. The study process and the PRISMA 2020 flowchart are shown in Figure 1.

The articles were published between 1988 and 2024 (the last search was conducted on 31 December 2024) and analysed various endogenous antioxidants which can indicate the intensity of oxidative stress in the body, as well as exogenous antioxidants and characteristic biomarkers of oxidative stress. The number, sex and age of the test subjects also varied. In 20 articles the exact number of male and female subjects is given, while in the other articles only the number of subjects per group is given. In 30 articles the exact age range of the test subjects is given, while in the other articles the mean value (average) is given. The studies included in the systematic review comprise investigations of various biomarkers of oxidative stress (particularly endogenous antioxidants) in individuals with DS. Of the 41 articles identified, 25 included an analysis based on a blood sample (including serum and plasma), while the remaining articles included an analysis based on a urine sample (5 articles), saliva (4 articles), amniotic fluid (4 articles), deciduous teeth (1 article), abdominal skin (1 article) and cerebral cortex (1 article). All of these biomarkers were included in the overview of data from the final selected studies (Supplementary File S1). Detailed summaries of the included articles can be found in Table 1, which lists the main components of each article (first author’s surname, year of publication, sex and age of subjects, indicator/oxidative stress factor studied, sample processed and the *p*-value for individual biomarkers/factors due to the significance of differences between subjects). Therefore, this article provides a systematic review of the current literature on the results of studies on biomarkers of oxidative stress in individuals with DS.

The results presented in Table 1 show a significant disturbance of the antioxidant balance and indicative oxidative damage in individuals with DS compared to the control group. Key alterations include increased SOD activity, decreased reduced glutathione (GSH) levels, and increased levels of malondialdehyde (MDA) and 8-hydroxy-2’-deoxyguanosine (8-OHdG). These changes therefore indicate a remarkable shift in the balance between oxidants and antioxidants in individuals with DS.

### 3.1. Enzymatic Antioxidants

It is known that excessive activity of the SOD1 in individuals with DS primarily contributes to increased oxidative stress. An imbalance in the activity of SOD and other essential antioxidant enzymes such as CAT and GPx can lead to an accumulation of H_2_O_2_, potentially resulting in damage to cellular structures. A well-known hypothesis is that the increased expression and activity of SOD1 due to DS occurs without a corresponding increase in the activities of CAT and GPx, which are critical for the effective degradation of H_2_O_2_ [31,46]. This oxidative imbalance is thought to be a primary pathophysiological mechanism contributing to the development of the disorders associated with DS. These include increased neurological dysfunction, cardiovascular diseases, the occurrence of haematological abnormalities, accelerated cellular ageing processes and an increased susceptibility to bacterial infections [7,46,94,96,97]. Many studies have shown that the activity of SOD in the blood of individuals with DS varies greatly [47,48,52,64]. This observation is consistent with the fact that the SOD1 gene is located on chromosome 21, which is upregulated by DS [98,99], and later studies have confirmed these findings [48,74]. In a study conducted by Garlet et al., an increased activity of SOD was found in serum samples from individuals with DS. Furthermore, CAT activity was significantly increased in these individuals compared to the control group. However, even the increased CAT activity does not appear to be sufficient to effectively neutralise the excess H_2_O_2_ resulting from the increased SOD1 activity, which further increases oxidative stress in individuals with DS [46]. In contrast to the other enzymes analysed, the activity of glutathione S-transferase (GST) was significantly lower in individuals with DS compared to the control group, indicating the presence of oxidative stress in these individuals [46,62]. The increased activity of SOD1 can lead to an accumulation of ·OH, which subsequently reduces the effectiveness of GST as part of the secondary antioxidant defence system [31,62]. Moreover, lower GST activity is associated with lower levels of GSH, an important non-enzymatic antioxidant that plays a crucial role in the neutralisation of H_2_O_2_ [46,63,66,100].

Besides blood and serum, saliva has also been recognised as a valuable biological sample for the assessment of oxidative stress in individuals with DS. Several studies have shown statistically significant differences in total protein (TP) content, total antioxidant status (TAS) and SOD activity in saliva of individuals with DS compared to the control group [82,83,84,85]. In recent years, a growing number of studies have investigated the biomarkers of oxidative stress in the amniotic fluid (AF) of pregnant women carrying a foetus with Ts21. The results of these studies indicate a significant increase in the activity of SOD, an increased content of oxidised proteins and an elevated content of products of lipid peroxidation. At the same time, the activity of important antioxidant enzymes such as CAT and GPx decreases significantly. This imbalance contributes to an increased overall level of oxidative damage [47,79,91,92]. These changes in biomarkers indicate the potential for early detection of oxidative stress in utero caused by different biological pathways [101]. The collected data indicate that oxidative stress during pregnancy in a foetus with Ts21 is a complex pathophysiological process that can significantly affect foetal development [79].

### 3.2. Non-Enzymatic Antioxidants

Non-enzymatic antioxidants such as GSH play an important role in defence against oxidative stress by rapidly protecting cellular integrity through various antioxidant mechanisms, but their levels can often be reduced in individuals with DS [33,81]. GSH is a potent non-enzymatic antioxidant for the detoxification of H_2_O_2_ and can influence elevated ROS levels both through direct interaction and indirectly by modulating the corresponding signalling pathways [63,66,100,102]. Studies indicate a significant correlation between decreased GSH levels and increased oxidative stress in individuals with DS [70,71,103]. Low GSH levels can impair the effectiveness of enzymatic antioxidants such as GST and GPx and thus contribute to elevated oxidative stress [46].

Analyses of blood and plasma samples have shown that individuals with DS have significantly lower GSH levels compared to control groups, indicating an impaired antioxidant defence system in this group [1,46,63]. It has also been shown that a progressive decline in GSH levels with increasing age, suggesting that oxidative stress may increase over time. In particular, older individuals with DS show a marked decline in GSH levels, which increases their susceptibility to oxidative damage. It was also found that TAS levels were significantly lower in individuals with DS compared to the control group, indicating a reduced capacity of the antioxidant system in this group. These findings suggest possible oxidative damage that may contribute to the pathophysiological processes associated with DS, including accelerated ageing and tissue damage [46,63,72,77]. In a study conducted by Campos et al., urine samples from individuals with DS aged between one and twelve years showed elevated uric acid (UA) levels compared to a control group of the same age [89]. This finding is consistent with the results of Garlet et al. who reported a significant increase in UA levels in the blood of individuals with DS aged up to 14 years compared to a control group [46]. The results also align with the study by Žitňanová et al. which showed that individuals with DS have significantly increased UA and allantoin (Alla) levels. Moreover, there is a positive correlation between these levels and the age of individuals with DS compared to controls, further supporting the notion of progressive ROS in individuals with DS [69]. The relatively high urate levels may indicate a compensatory antioxidant response to the prolonged oxidative stress associated with DS. In contrast, no statistically significant differences in UA concentrations were found between the DS group and the corresponding control group in adults aged 43 to 61 years [1,46,88].

### 3.3. Biomarkers of Oxidative Damage

Individuals with DS have an increased susceptibility to reactive ROS, which can cause damage that leads to degenerative changes in various tissues, including the brain, heart, eyes and thyroid [72,73,75,78]. As a result, there is a significant increase in lipid and protein peroxidation, characterised by increased MDA levels in the erythrocytes, and this increase can impair the function of various enzymes [68,76]. The increase in lipid peroxidation is also associated with higher levels of ROS and RNS, indicating a disturbed homeostatic balance in oxidative metabolism in individuals with DS. Due to its high chemical reactivity, MDA can cause additional structural and functional changes in the cells [65]. Elevated levels of MDA and protein carbonyls (PCs) have been found in the plasma of individuals with DS, which has been confirmed by several previous studies [67,103,104]. These biomarkers can have a negative effect on enzyme activities and disrupt the structure and function of cell membranes, ultimately leading to a change in cellular homeostasis. Oxidative damage to proteins is of particular concern as it can lead to changes in their structure, enzyme activity and signalling pathways, ultimately contributing to accelerated ageing and the development of various diseases [72,81,90]. Interestingly, there were also no significant differences in the plasma levels of 4-hydroxynonenal (4-HNE) between individuals with DS and those without DS [80].

Saliva has also proven to be an effective sample for the assessment of oxidative stress. Studies have shown significantly elevated levels of MDA, PCO and 8-OHdG in the saliva of individuals with DS [82,83,85]. In particular, 8-OHdG concentrations were higher in adults with DS (over 30 years old) than in age-matched controls and younger individuals with DS (1–12 years old). The results suggest increased oxidative stress activity in the saliva of individuals with DS, possibly indicating pathological processes associated with increased oxidative stress in this aneuploidy, such as accelerated ageing. This observation is consistent with the finding that progressive oxidative stress can occur in individuals with DS [85]. In studies conducted by Campos et al. in 2010 and 2011, they analysed the concentrations of specific biomarkers for oxidative stress in the urine of younger and older individuals with and without DS [88,89]. This is in line with previous studies that have shown that the urine of younger individuals often contains elevated levels of biomarkers associated with oxidative stress [88,105,106]. In their 2011 study, Campos et al. observed elevated levels of 8-OHdG and dityrosine (diTyr) in younger individuals with DS (under 10 years of age) compared to a control group. Biomarkers such as F_2_-isoprostane (F_2_-isoPs), thiobarbituric acid-reacting substances (TBARS) and advanced glycation end products were also significantly elevated in this younger group, with some biomarkers showing a negative correlation with age. In contrast, no significant differences between the levels of F_2_-isoPs and TBARS were found between the group with DS and the control group in the older individuals. It is worth noting that significantly higher diTyr levels were consistently found in the urine of individuals with DS in all age groups compared to controls [87]. In contrast, a study by Toluna et al. analysing Alla and F_2_-isoPs as biomarkers of oxidative stress found no significant increase in oxidative stress in individuals with DS compared to controls [86]. These results indicate that, contrary to previous assumptions, individuals with DS do not necessarily have elevated levels of oxidative stress when analysed using these specific biomarkers. These discrepancies are likely due to the heterogeneity associated with certain biomarkers and assays. Biomarkers of DNA and protein oxidation, such as 8-OHdG and diTyr, reflect redox pathways that show only a weak correlation with the urate- and lipid-based biomarkers analysed, including Alla and 2,3-dinor-8-iso-prostaglandin F_2α_-III (2,3-dinor-iPF_2α_-III). Studies have shown that the agreement between these different methods is only modest. Furthermore, the age-related compensatory upregulation of antioxidants and repair mechanisms observed in individuals with DS may normalise signs of systemic lipid peroxidation, although certain DNA and protein adducts may remain elevated. Therefore, it is likely that pathway- and age-specific effects contribute to the seemingly contradictory results [91,107,108].

Significant differences in oxidative status were also observed during the prenatal period when it comes to DS. The analysis performed by Odetti et al. revealed elevated levels of lipid and protein oxidation in foetuses with Ts21 compared to a control group. The concentration of glycation products, especially AGEs, was increased. These are substances that can lead to tissue and organ damage and are associated with the development of various diseases. These results indicate that significant oxidative stress occurs in the brains of foetuses with Ts21, suggesting that this stress will be felt throughout their lives. Oxidative stress has the potential to damage cell structure and function, which may contribute to the neurological complications typically associated with DS [95].

### 3.4. Inflammatory and Neurological Biomarkers Associated with Oxidative Stress

Previous studies have highlighted that the incidence of infectious and autoimmune diseases is significantly elevated in individuals with DS, regardless of factors such as sex, age, family history or other risk variables [109]. A study by Tarani et al. from 2020 [5] specifically investigated neuroinflammatory biomarkers in the serum of prepubertal individuals with DS (aged between one year and nine years and six months), focusing on nerve growth factor (NGF), brain-derived neurotrophic factor (BDNF), oxidative free radical defence (FORD) indicators, oxygen free radical (FORT) levels and cytokines, all of which play a crucial role in neuroinflammation and oxidative stress processes. The results showed no significant differences in NGF, FORD and FORT levels when comparing individuals with DS to the control group. However, the analysis revealed a significant increase in BDNF levels and a remarkable decrease in all analysed cytokines in individuals with DS compared to the control group. Furthermore, statistically significant sex-specific differences were observed in the serum cytokine levels of individuals with DS. This study emphasises the importance of investigating neuroinflammatory processes in DS in order to develop targeted strategies for the early detection and possible treatment of inflammatory conditions in these individuals [5].

Sun et al. explored the relationship between dopamine-related oxidative stress and mitochondrial dysfunction in dopaminergic neurons of individuals with DS (age six to ten years). Their analysis of dopaminergic neurons differentiated from deciduous teeth-derived stem cells of these individuals suggests that dysregulation of dopamine homeostasis may contribute to oxidative stress and mitochondrial dysfunction in the dopaminergic system of individuals with DS. This study emphasises the importance of understanding the specific biological changes that occur in the dopamine neurons of individuals with DS. The identification of increased oxidative stress and mitochondrial dysfunction provides the basis for further studies focussing on the development of targeted therapies or interventions to improve the neurological prognosis and overall quality of life of individuals with DS [93].

### 3.5. Metabolic Mediators of Oxidative Stress

Studies from 2016 were among the first to reveal that citrate metabolism contributes significantly to the oxidative stress observed in individuals with DS [110,111]. Accordingly, Convertini et al. found that individuals with DS aged three to five years had higher citrate levels compared to a control group of healthy individuals of the same age. The increased concentrations of citrate entering the cytosol from the mitochondria have been shown to promote the production of reactive species, including ROS and RNS, and thus increase the oxidative load on the cells [61]. Namely, citrate serves as a source of acetyl units for lipid biosynthesis, and its breakdown produces oxaloacetate, which is a precursor for the synthesis of nicotinamide adenine dinucleotide phosphate (NADPH) [111,112]. Since NADPH is a crucial reducing agent for the enzyme-mediated generation of ROS and RNS by NADPH oxidase and inducible nitric oxide synthase (iNOS), its role is particularly important. Nevertheless, the exact molecular mechanisms that lead to elevated citrate levels in individuals with DS are not yet fully understood [61].

Recent studies have also found elevated levels of asprosin, a relatively new antioxidant protein, in the plasma and AF of pregnant women carrying foetuses diagnosed with Ts21 [79,113]. Asprosin plays several vital roles in the body, particularly in the regulation of glucose metabolism, cell apoptosis, appetite and the modulation of central and peripheral nervous system functions [79,114]. The increased presence of asprosin in pregnancies with a foetus affected by Ts21 could represent an adaptive response to the oxidative stress that presumably occurs during intrauterine development [79,113,115]. Over time, a growing number of studies have investigated the biomarkers of oxidative stress in the AF of pregnant women carrying a foetus with Ts21. The elevated levels of asprosin observed in these pregnancies support the hypothesis that foetuses with Ts21 experience oxidative disturbances in the early stages of development. This emphasises the hypothesis of early onset and accumulation of oxidative damage that may have clinical implications for life after birth [47,79,91,92].

## 4. Conclusions

This article provides a systematic review of studies on biomarkers of oxidative stress in individuals with DS, with a particular focus on endogenous antioxidants. The analysis of the selected studies shows that the development of oxidative stress in individuals with DS is a complex process caused not only by an imbalance of SOD activity or overexpression of certain genes on chromosome 21, but also by other, less explored sources (e.g., high citrate levels, mitochondrial dysfunction, inflammatory processes, etc.). Indicators of oxidative stress, including antioxidants and biomarkers, are analysed using a variety of methods and samples. Despite extensive studies and a considerable amount of data on oxidative stress in DS, there are still notable limitations on this topic. The heterogeneity of study populations, particularly in terms of age, sex and methodological approaches, poses a challenge to the interpretation of these results. The standardisation of biomarkers of oxidative stress in DS and the uniform application of experimental protocols across studies are essential prerequisites for drawing definitive and valid conclusions. Longitudinal studies that track changes in oxidative stress biomarkers over time, especially as individuals with DS age, are essential for a better understanding of the progression of oxidative damage and its relationship to DS-related pathologies as well as other oxidative stress-related diseases common in this aneuploidy. Subgroup analyses targeting specific comorbidities or age groups may help to identify tailored therapeutic approaches. Future studies should emphasise the clinical potential of antioxidant therapies and other strategies to reduce oxidative stress in DS to improve quality of life and prolong life expectancy.

## Figures and Tables

**Table 1:** Summary of the selected articles.

				Subjects		Controls		*p*-Value
First Author Year	Objective of the Study	Indicator	Specimen	N (M/F)	AGE	N (F/M)	AGE	
Convertini 2016 [61]	to evaluate the contribution of the citrate pathway to oxidative stress	citrate	heparinized blood	10	3–5 years	10	3–5 years	<0.05
		ATP-citrate lyase (ACLY)						<0.001
		citrate carrier (CIC)						<0.001
		ROS						<0.01
		NO						<0.05
		lipid peroxidation						<0.05
Ferreira 2015 [52]	to evaluate the activities of ectonucleotidases in the blood platelets of DS individuals	adenosine monophosphate (AMP)	blood	28 (12 F and 16 M)	26.20 ± 5.76 (F) years 28.88 ± 6.94 (M) years	28 (15 F and 13 M)	24.12 ± 5.54 (F) years 23.67 ± 3.98 (M) years	<0.05
		adenosine deaminase (ADA)						<0.05
		adenosine triphosphate (ATP)						<0.05
		adenosine monophosphate (ADP)						N.S.
		lipid peroxidation						<0.05
		sulfhydryl content						<0.05
		superoxide dismutase (SOD)						<0.05
		catalase activity						<0.05
Sadiq 2015 [62]	to investigate the possible association between antioxidant/redox status and DNA instability in DS individuals	superoxide dismutase (SOD)	blood	19	5–16 years	19	5–16 years	0.042
		catalase (CAT)						N.S.
		glutathione peroxidase (GPx)						N.S.
		glutathione S-transferases (GST)						0.007
Garlet 2013 [46]	to assess the antioxidant status and oxidative stress biomarkers in the blood of individuals with DS	superoxide dismutase (SOD)	blood	20	3–14 years	18 (8 F and 10 M)	3–12 years	<0.001
		catalase (CAT)						<0.001
		glutathione peroxidase (GPx)						N.S.
		glutathione reductase (GR)						<0.001
		reduced glutathione (GSH)						<0.05
		glutathione S-transferases (GST)						<0.001
		uric acid (UA)						<0.05
		protein carbonyls (PCs)						<0.05
Sulthana 2012 [63]	to investigate oxidative stress in individuals with DS by determining the levels of non-enzymatic antioxidants such as reduced glutathione and total antioxidant status	reduced glutathione (GSH)	blood	19	0–4 years	19	0–4 years	N.S.
				6	4–8 years	6	4–8 years	N.S.
				6	>8 years	6	>8 years	<0.05
Sulthana 2012 [64]	to evaluate the activity of enzymatic antioxidants in individuals with DS	superoxide dismutase (SOD)	blood	31 (13 F and 18 M)	3 months–14 years	31 (13 F and 18 M)	3 months–14 years	N.S.
		catalase (CAT)						N.S.
		glutathione peroxidase (GPx)						N.S.
		SOD 1/CAT + GPx						<0.05
Casado 2007 [65]	to detect a change in malondialdehyde levels due to oxidative stress	malondialdehyde (MDA)	blood	100 (66 F and 34 M)	0–29 years	100 (60 F and 40 M)	0–29 years	<0.05
Pallardó 2006 [1]	evaluate a set of biomarkers of oxidative stress in DS individuals that could provide in vivo evidence of their propensity for accelerated ageing and other redox-related pathologies that occur in DS individuals	8-hydroxy-2’-deoxyguanosine (8-OHdG)	blood	32 (18 F and 14 M)	2 months–57 years	67	2 months–57 years	
					1–10 years		1–10 years	0.022
					11–20 years		11–20 years	0.0003
					21–30 years		21–30 years	0.069
					31–57 years		31–57 years	N.S.
		total glutathione (GSHt)			<15 years		<15 years	N.S.
					>15 years		>15 years	0.05
		glutathione disulfide (GSSG)			<15 years		<15 years	0.006
					>15 years		>15 years	N.S.
		reduced glutathione (GSH)			<15 years		<15 years	N.S.
					>15 years		>15 years	0.05
		GSSG/GSH × 100			<15 years		<15 years	0.049
					>15 years		>15 years	0.0003
		glyoxal (Glx)			<15 years		<15 years	0.003
					>15 years		>15 years	N.S.
		methylglyoxal (MGlx)			<15 years		<15 years	N.S.
					>15 years		>15 years	0.008
		uric acid (UA)			<15 years		<15 years	0.013
					>15 years		>15 years	0.016
		xanthine oxidase (XO)						0.008
Ordonez 2006 [66]	to determine the activity of glucose-6-phosphate dehydrogenase in individuals with DS in order to analyse its role as a minimally invasive bioindicator of oxidative damage	superoxide dismutase (SOD)	blood	31 M	16.3 ± 1.1 years	17	16.6 ± 1.3 years	0.019
		glutathione peroxidase (GPx)						0.030
		catalase (CAT)						N.S.
		glucose-6-phosphate dehydrogenase (G6PDH)						0.038
Garcez 2005 [67]	to assess the levels of thiobarbituric acid reactive substances, uric acid and seric superoxide dismutase and catalase activities as well as serum total iron, total iron binding capacity (TIBC), erythrocyte osmotic fragility and haemograms in individuals with DS	thiobarbituric acid-reacting substances (TBARS)	blood	50 (25 F and 25 M)	3–24 years	50 (25 F and 25 M)	3–24 years	
				F		F		0.002
				M		M		0.047
				F + M		F + M		0.002
		superoxide dismutase (SOD)		F		F		0.001
				M		M		0.001
				F + M		F + M		0.004
		catalase (CAT)		F		F		0.005
				M		M		0.002
				F + M		F + M		0.002
		uric acid (UA)		F		F		0.001
				M		M		0.001
				F + M		F + M		0.001
Garaiová 2004 [68]	to investigate the relationship between the ratio of the activities of the antioxidant enzymes R = SOD/(GPx + CAT) and the content of non-enzymatic low molecular weight antioxidants (LMWA) (reduced and oxidised glutathione, vitamin E, uric acid, total antioxidant status) as well as the concentrations of malondialdehyde in erythrocytes and lipofuscin in the serum of individuals with DS	superoxide dismutase (SOD)	blood	44	23.208 ± 1.967 years	26	23.340 ± 2.978 years	<0.001
		glutathione peroxidase (GPx)						<0.001
		catalase (CAT)						N.S.
		reduced glutathione (GSH)						0.064
		glutathione disulfide (GSSG)						0.012
		GSH/GSSG						N.S.
		vitamin E						
		uric acid (UA)						0.007
		total antioxidant status (TAS)						0.031
		malondialdehyde (MDA)						0.019
		lipofuscin						N.S.
		erythrocytes R = SOD/(GPx + CAT)						0.006
Žitňanová 2004 [69]	to compare the levels of purine metabolites (uric acid, hypoxanthine and xanthine) in the plasma of DS individuals with that of healthy control subjects and to analyse the levels of allantoin in both groups	uric acid (UA)	blood	16	10.06 ± 1.04 years	16	11.94 ± 0.97 years	<0.05
		hypoxanthine (HX)						<0.05
		xanthine (X)						<0.05
		allantoin (Alla)						<0.05
Pastor 2003 [70]	to evaluate the concentrations of all forms of glutathione, including glutathionyl haemoglobin, as well as the enzyme activities of superoxide dismutase, glutathione peroxidase, glutathione reductase and glutathione S-transferase in the blood of individuals with DS	total glutathione (GSHt)	blood	46 (26 F and 20 M)	6.7 ± 2.7 years	64 (34 F and 30 M)	5.1 ± 2.3 years	<0.0001
		free glutathione (GSHf)						<0.0001
		GSSG/GSH						<0.0001
		glutathionyl-haemoglobin (GS-Hb)						<0.0001
		superoxide dismutase (SOD)						<0.0001
		glutathione peroxidase (GPx)						<0.05
		SOD/GPx						<0.0001
		glutathione reductase (GR)						N.S.
		glutathione S-transferase (GST)						<0.05
Pogribna 2001 [71]	to evaluate the effects of overexpression of the cystathionine beta-synthase (CBS) gene on homocysteine metabolism in individuals with DS and to determine whether supplementation of Ts21 lymphoblasts in vitro with selected nutrients would shift the genetically determined metabolic imbalance	total homocysteine (tHcy)	blood	42	7.4 ± 4.2 years	36	7.4 ± 4.2 years	<0.001
		methionine (Met)						<0.001
		cystathionine						<0.001
		cysteine (CYS)						<0.001
		reduced glutathione (GSH)						<0.001
		S-adenosylmethionine (SAM)						<0.04
		S-adenosylhomocysteine (SAH)						<0.04
		adenosine						<0.001
Kanavin 2000 [72]	to investigate the hypothesis of a role of an imbalance between the production of toxic oxygen and protective metallo-enzymes in the development of hypothyroidism in DS individuals	free thyroxine (fT4)	blood	38 (22 F and 16 M)	33 ± 11.1 years	39 (22 F and 17 M)	33 ± 11.1 years	<0.05
		total thyroxine (tT4)						<0.05
		thyroid stimulating hormone (TSH)						<0.05
		triiodothyronine (T3)						N.S.
		thyroxine-binding globulin (TBG)						<0.05
		high-density lipoprotein (HDL)						<0.05
		low-density lipoprotein (LDL)						N.S.
		triglycerides (TGs)						<0.05
Brugge 1999 [73]	to investigate the possible relationship between biochemical indices of free radical metabolism (FRM) and specific memory deficits in individuals with DS	copper-zinc superoxide dismutase (SOD1)	blood	17 (14 F and 3 M)	22–51 years	11 (5 F and 6 M)	22–48 years	<0.02
		glutathione peroxidase (GPx)						<0.01
		catalase (CAT)						N.S.
Pastor 1998 [74]	to evaluate the cellular antioxidant system by determining the catalytic activity of the enzymes copper-zinc superoxide dismutase, glutathione peroxidase, catalase and glutathione reductase as well as the concentrations of alpha-tocopherol in the erythrocytes of DS individuals	copper-zinc superoxide dismutase (SOD1)	blood	72	17.8 ± 15.8 years	72	14.6 ± 10.8 years	0.0001
		glutathione peroxidase (GPx)						0.0001
		catalase (CAT)						0.026
		glutathione reductase (GR)						0.0001
Gerli 1990 [75]	to evaluate the level of antioxidant enzyme activities copper-zinc superoxide dismutase, catalase, glutathione peroxidase and reduced glutathione in the erythrocytes of individuals with DS	copper-zinc superoxide dismutase (SOD1)	blood	39 (18 F and 21 M)	14–53 years	50 (25 F and 25 M)	23–60 years	<0.001
		catalase (CAT)						N.S.
		glutathione peroxidase (GPx)						<0.001
		reduced glutathione (GSH)						N.S.
Bras 1989 [76]	to obtain further evidence for the possible role of increased superoxide dismutase activity in oxidative damage in individuals with DS	thiobarbituric acid (TBA)	blood	9	9 months–22 years	9	9 months–22 years	<0.01
Lazzarino 2022 [77]	distinguish the potential effects of ageing from those of pathobiological processes associated with DS on circulating levels of the aforementioned compounds, identify the metabolic pathways that are actually altered by DS, recognise certain biomarkers that are unique to DS and therefore useful to drive future potential DS-targeted pharmacological treatments	aspartate (Asp)	serum	YDSP = 29 (13 F and 16 M) ADSP = 27 (12 F and 15 M)	20–40 years (YDSP) 41–60 years (ADSP)	YnonDSP = 55 (26 F and 29 M) AnonDSP = 47 (22 F and 25 M)	30–60 years (YnonDSP) 75–90 years (AnonDSP)	<0.0001
		glutamate (Glu)						<0.0001
		asparagine (Asn)						<0.0001
		serine (Ser)						<0.0001
		glutamine (Gln)						<0.0001
		histidine (His)						<0.0001
		glycine (Gly)						N.S.
		threonine (Thr)						<0.0001
		citrulline (Cit)						<0.02
		arginine (Arg)						<0.0001
		alanine (Ala)						N.S.
		taurine (Tau)						<0.0001
		tyrosine (Tyr)						<0.01
		valine (Val)						N.S.
		methionine (Met)						<0.0001
		tryptophan (Trp)						<0.0001
		phenylalanine (Phe)						<0.0001
		isoleucine (Ile)						N.S.
		leucine (Leu)						<0.02
		ornithine (Orn)						<0.0001
		lysine (Lys)						N.S.
		uracil						<0.0001
		beta-pseudouridine						<0.0001
		uridine						<0.0001
		hypoxanthine						<0.0001
		xanthine						<0.0001
		uric acid (UA)						<0.0001
		sum of oxypurines						<0.0001
		inosine						<0.0001
		vitamin C						<0.002
		reduced glutathione (GSH)						<0.001
		nitrites						<0.002
		nitrates						<0.0001
		nitrites + nitrates						<0.0001
		lactate						<0.0001
		creatinine (Cr)						N.S.
Tarani 2020 [5]	to determine and correlate serum levels of nerve growth factor and brain neurotrophic factor in prepubertal male and female DS individuals (i); (ii) to measure oxidative status in serum as an oxygen free radical defence and oxygen free radical assay; and (iii) the serum levels of cytokines that play a subtle role in both neuroinflammatory and oxidative processes, such as tumour necrosis factor alpha, transforming growth factor beta, monocyte chemoattractant protein-1, interleukin-1 alfa, interleukin-2, interleukin-6, interleukin-10, interleukin-12	nerve growth factor (NGF)	serum	9 (4 F and 5 M)	1–9.6 years	21 (11 F and 10 M)	1–9.6 years	N.S.
		brain-derived neurotrophic factor (BDNF)						<0.05
		free oxygen radicals defense (FORD)						N.S.
		free oxygen radicals test (FORT)						N.S.
		tumour necrosis factor alpha (TNF-α)						N.S.
		transforming growth factor beta (TGF-β)						N.S.
		monocyte chemoattractant protein-1 (MCP1)						N.S.
		interleukin-1 alpha (IL-1α)						<0.01
		interleukin-2 (IL-2)						<0.01
		interleukin-6 (IL-6)						<0.01
		interleukin-10 (IL-10)						<0.01
		interleukin-12 (IL-12)						<0.01
Manna 2016 [78]	to investigate a possible pathogenic role of iron in neurodegeneration	total iron serum (STI)	serum	16	18–35 years	16	18–35 years	N.S.
		ferritin						0.0093
		transferrin						0.0093
Buczyńska 2021 [79]	assessment of the utility of selected parameters of oxidative stress biomarkers in maternal plasma and amniotic fluid for DS screening	25-hydroxyvitamin D (25(OH)D)	plasma	20 F	15–18 weeks of gestation	20 F	15–18 weeks of gestation	N.S.
		aspros						<0.0001
		advanced glycation end products (AGEs)						<0.001
		ischemia-modified albumin (IMA)						<0.0001
		alpha-1-antitrypsin (A1AT)						N.S.
		DNA/RNA oxidative stress damage products (OSDP)						N.S.
Manna 2016 [78]	to investigate a possible pathogenic role of iron in neurodegeneration	uric acid (UA)	plasma	16	18–35 years	16	18–35 years	0.0013
		plasma nonprotein-bound iron (P-NPBI)						0.0013
		intraerythrocyte non-protein bound iron (IE-NPBI)						0.0004
		ROS						0.0004
		F_2_-isoprostane (F_2_-isoPs)						0.0001
		F_4_-neuroprostanes (F_4_-NeuroPs)						0.0032
		F_2_-dihomo-isoprostanes (F_2_-dihomo-IsoPs)						0.0001
Garlet 2013 [46]	to assess the antioxidant status and oxidative stress biomarkers in the blood of individuals with DS	vitamin E	plasma	20	3–14 years	18 (8 F and 10 M)	3–12 years	N.S.
		thiobarbituric acid-reacting substances (TBARS)						N.S.
Sulthana 2012 [63]	to investigate oxidative stress in individuals with DS by estimating the levels of non-enzymatic antioxidants such as reduced glutathione and total antioxidants status	total antioxidant status (TAS)	plasma	19	0–4 years	19	0–4 years	<0.05
				6	4–8 years	6	4–8 years	N.S.
				6	>8 years	6	>8 years	N.S.
Sulthana 2012 [64]	assessment of oxidative stress in DS by determining products of oxidative damage such as plasma malondialdehyde and plasma protein carbonylation	malondialdehyde (MDA)	plasma	19	3 months–4 years	19	3 months–4 years	0.0002
				6	4–8 years	6	4–8 years	0.0102
				6	>8 years	6	>8 years	N.S.
		protein carbonylation (PCO)		19	0–4 years	19	0–4 years	<0.0001
				6	4–8 years	6	4–8 years	0.0065
				6	8–14 years	6	8–14 years	0.0022
Žitňanová 2006 [80]	to evaluate the role of oxidative stress in individuals with DS and to investigate the effects of an imbalance of antioxidant enzyme activities in individuals with DS on the formation of oxidative stress biomarkers	protein carbonyls (PCs)	plasma	20	10.06 ± 1.04 years	18	11.94 ± 0.97 years	0.002
		ferric reducing ability of plasma (FRAP)						N.S.
		4-hydroxynonenal (4-HNE)						N.S.
Pinto 2002 [81]	glutathione and other lesser-known antioxidant mechanisms to determine if there are changes in reactive oxygen species in individuals with DS	reduced glutathione (GSH)	plasma	60	0.5–12 years	29	1–17 years	N.S.
		glutathione disulfide (GSSG)						N.S.
		total glutathione (GSHt)						N.S.
		acid phosphatase (ACP1)						N.S.
		methemoglobin reductase (MHR)						N.S.
		transmembrane reductase (TMR)						N.S.
Bras 1989 [76]	to obtain further evidence for the possible role of increased copper-zinc superoxide dismutase activity in oxidative damage in individuals with DS	thiobarbituric acid (TBA)	plasma	9	9 months–22 years	9	9 months–22 years	N.S.
		uric acid (UA)						N.S.
		vitamin C						N.S.
		vitamin E						N.S.
Domingues 2017 [82]	to correlate clinical parameters with salivary parameters and the content of cariogenic and periodontopathogenic bacteria and to evaluate the antioxidant profile in individuals with DS compared to individuals without DS	total protein (TP)	saliva	18		23		<0.0001
		glutathione peroxidase (GPx)						N.S.
		superoxide dismutase (SOD)						0.0002
		total antioxidant capacity of saliva (TAOC)						N.S.
		malondialdehyde (MDA)						<0.001
de Sousa 2015 [83]	investigation of the enzymatic and non-enzymatic antioxidant systems and the levels of biomarkers for oxidative damage in the saliva of individuals with DS	superoxide dismutase (SOD)	saliva	30	14–24 years	30	14–24 years	<0.05
		total protein (TP)						<0.05
		carbonylated proteins						<0.05
		uric acid (UA)						N.S.
		vitamin C						N.S.
		peroxidase						N.S.
		total antioxidant status (TAS)						N.S.
Subramaniam 2014 [84]	to assess the total antioxidant status, nitric oxide and sialic acid of saliva in individuals with DS and their relationship to their oral health status	total antioxidant status (TAS)	saliva	34 (19 F and 15 M)	9.44 ± 1.50 years	34 (13 F and 21 M)	9.29 ± 1.98 years	0.001
		nitric oxide (NO)						N.S.
		sialic acid (SA)						0.001
Komatsu 2013 [85]	evaluation of 8-hydroxy-2’-deoxyguanosine as a marker for oxidative stress in the saliva of DS individuals	8-hydroxy-20-deoxyguanosine (8-OHdG)	saliva	45 (24 F and 21 M)	1–12 years	45 (22 F and 23 M)	1–12 years	<0.01
				21 (3 F and 18 M)	30–66 years	26 (20 F and 6 M)	30–58 years	<0.01
Tolun 2012 [86]	comparison of urinary levels of allantoin and 2,3-dinor-iPF_2a_-III in DS individuals and control subjects	allantoin (Alla)	urine	48 (23 F and 25 M)	2–52 years	130 (71 F and 59 M) —Alla	4–78 years	<0.05
		2,3-dinor-8-iso-prostaglandin F_2α_-III (2,3-dinor-iPF_2α_-III)				85 (49 F and 36 M) —2,3-dinor-iPF_2ɑ_-III	4–75 years	N.S.
Campos 2011 [87]	assess a comprehensive set of urinary biomarkers of oxidative/nitrosative stress in individuals with and without DS	creatinine (Cr)	urine	26 (13 F and 13 M)	3–14 years	19 (11 F and 8 M)	5–14 years	N.S.
		8-hydroxy-2’-deoxyguanosine (8-OHdG)						N.S.
		isoprostane (15-F_2t_-IsoP)						N.S.
		thiobarbituric acid-reacting substances (TBARS)						N.S.
		advanced glycation end product (AGEs)						N.S.
		dityrosine (diTyr)						<0.05
		hydrogen peroxide (H_2_O_2_)						N.S.
		total nitrite and nitrate (tNO_x_)						N.S.
Campos 2011 [88]	to evaluate a comprehensive set of urinary biomarkers of oxidative/nitrosative stress in individuals with and without DS	creatinine (Cr)	urine	78 (43 F and 35 M)	15–59 years	65 (39 F and 26 M)	15–59 years	<0.001
		8-hydroxy-2’-deoxyguanosine (8-OHdG)						N.S.
		isoprostane (15-F_2t_-IsoP)						N.S.
		thiobarbituric acid-reacting substances (TBARS)						N.S.
		advanced glycation end product (AGEs)						N.S.
		dityrosine (diTyr)						<0.001
		hydrogen peroxide (H_2_O_2_)						<0.001
		total nitrite and nitrate (tNO_x_)						<0.001
Campos 2010 [89]	comparison of uric acid levels and antioxidant status in a sample of individuals with DS with those of healthy, age-matched controls to assess the role of oxidative stress in these individuals	creatinine (Cr)	urine	19 (6 F and 13 M)	1–12 years	14 (8 F and 6 M)	5–13 years	N.S.
				13 (6 F and 7 M)	43–57 years	15 (10 F and 5 M)	43–61 years	N.S.
		total antioxidant status (TAS)/creatinine (Cr)		19 (6 F and 13 M)	1–12 years	14 (8 F and 6 M)	5–13 years	0.015
				13 (6 F and 7 M)	43–57 years	15 (10 F and 5 M)	43–61 years	N.S.
		total antioxidant status without relative contribution of uric acid (TAS−UA)/creatinine (Cr)		19 (6 F and 13 M)	1–12 years	14 (8 F and 6 M)	5–13 years	N.S.
				13 (6 F and 7 M)	43–57 years	15 (10 F and 5 M)	43–61 years	0.033
		uric acid (UA)/creatinine (Cr)		19 (6 F and 13 M)	1–12 years	14 (8 F and 6 M)	5–13 years	0.045
				13 (6 F and 7 M)	43–57 years	15 (10 F and 5 M)	43–61 years	N.S.
Jovanovic 1998 [90]	to assess the role of oxidative stress in DS	8-hydroxy-2’-deoxyguanosine (8-OHdG)	urine	85	5.11 ± 4.15 years	81	7.56 ± 4.67 years	0.00011
		thiobarbituric acid-reacting substances (TBARS)						0.033
Buczyńska 2021 [79]	assessment of the utility of selected parameters of oxidative stress biomarkers in maternal plasma and amniotic fluid for DS screening	25-hydroxyvitamin D (25(OH)D)	amniotic fluid	20 F	15–18 weeks of gestation	20 F	15–18 weeks of gestation	N.S.
		aspros						<0.0001
		advanced glycation end products (AGEs)						<0.001
		ischemia-modified albumin (IMA)						<0.0001
		alpha-1-antitrypsin (A1AT)						N.S.
		DNA/RNA oxidative stress damage products (OSDP)						N.S.
Bahsi 2022 [47]	to assess the status of the oxidant/antioxidant system and the levels of interleukin-6/interleukin-10 in the amniotic fluid of expectant mothers carrying a child with Ts21 identified by amniocentesis	catalase (CAT)	amniotic fluid	18	16–20 weeks of gestation	13	16–20 weeks of gestation	0.034
		malondialdehyde (MDA)						N.S. (0.323)
		superoxide dismutase (SOD)						0.012
		glutathione peroxidase (GPx)						N.S. (0.566)
		adenosine deaminase (ADA)						N.S. (0.149)
		xanthine oxidase (XO)						N.S. (0.114)
		nitric oxide (NO)						N.S. (0.749)
		nitric oxide synthase (NOS)						N.S. (0.434)
		interleukin-6 (IL-6)						0.017
		interleukin-10 (IL-10)						N.S. (0.425)
Buczyńska 2021 [79]	assessment of the utility of selected parameters of oxidative stress biomarkers in maternal plasma and amniotic fluid for DS screening	25-hydroxyvitamin D (25(OH)D)	amniotic fluid	20 F	15–18 weeks of gestation	20 F	15–18 weeks of gestation	N.S.
		aspros						<0.0001
		advanced glycation end products (AGEs)						<0.0001
		ischemia-modified albumin (IMA)						N.S.
		alpha-1-antitrypsin (A1AT)						<0.0001
		DNA/RNA oxidative stress damage products (OSDP)						<0.05
Perluigi 2011 [91]	evaluate a set of biomarkers of oxidative stress in the amniotic fluid of women carrying foetuses with Ts21 that could detect the early onset of oxidative damage in Ts21 in vivo	protein carbonylation (PCO)	amniotic fluid	10 F	15–17 weeks of gestation	10 F	15–17 weeks of gestation	<0.05
		4-hydroxynonenal (4-HNE)						<0.05
		thioredoxin (Trx)						<0.05
		total glutathione (GSHt)						<0.05
		glutathione disulfide (GSSG)						<0.05
Perrone 2007 [92]	to test the hypothesis that oxidative stress occurs early in pregnancies with foetuses with Ts21	isoprostane (IP)	amniotic fluid	10 F	16th week of gestation	56 F	16th week of gestation	<0.0001
Sun 2022 [93]	to further elucidate the pathology of dopaminergic neurons (DNs) in DS	dopamine in dopaminergic neurons (DN)	stem cells from exfoliated baby teeth	3 (M)	6, 9 and 10 years	3 (3 M)	6, 6 and 7 years	<0.001
		ROS						<0.001
Rodríguez-Sureda 2015 [94]	to determine whether there is an imbalance in the activities, messenger ribonucleic acid (mRNA) and protein expression of the antioxidant enzymes copper-zinc superoxide dismutase, superoxide dismutase-2 (SOD2), glutathione peroxidase and catalase during the cell replication process in vitro	oxidised proteins	abdominal skin	5	9–22 weeks of gestation	5	9–22 weeks of gestation	<0.05
		malondialdehyde (MDA)						<0.01
		superoxide dismutase (SOD1)						<0.01–<0.001
		catalase (CAT)						<0.05–<0.01
		glutathione peroxidase (GPx)						N.S.–<0.05
Odetti 1998 [95]	to further investigate the issue of oxidative stress through the presence and amount of lipid and protein oxidation biomarkers in the foetal cortex of DS	protein carbonyls (PCs)	the cerebral cortex	8	18–20 weeks of gestation	4	18–20 weeks of gestation	<0.05
		thiobarbituric acid-reacting substances (TBARS)						<0.05
		4-hydroxynonenal (4-HNE)						<0.05
		pyrraline						<0.05
		pentosidine						<0.05

## Supplementary Materials

The following supporting information can be downloaded at: https://www.mdpi.com/article/10.3390/antiox14070816/s1, Supplementary File S1: Analysed Values from Body Fluids (Blood Except Serum and Plasma, Serum, Plasma, Saliva, Urine and Amniotic Fluid).

## Abbreviations, Acronyms and Initialisms

The following abbreviations, acronyms and initialisms are used in this manuscript:

%	percentage
/	slash
+	plus
<	less than
=	equal to
>	greater than
±	plus-minus
×	multiplication
·OH	hydroxyl radical
15-F_2t_-IsoP	isoprostane
2,3-dinor-iPF_2α_-III	2,3-dinor-8-iso-prostaglandin F_2α_-III
25(OH)D	25-hydroxyvitamin D
4-HNE	4-hydroxynonenal
8-OHdG	8-hydroxy-2’-deoxyguanosine
α	alpha
β	beta
A1AT	alpha-1-antitrypsin
ACLY	ATP-citrate lyase
ACP1	acid phosphatase
ADA	adenosine deaminase
ADP	adenosine monophosphate
ADSP	aged Down syndrome patients
AF	amniotic fluid
AGEs	advanced glycation end products
Ala	alanine
Alla	allantoin
AMP	adenosine monophosphate
AnonDSP	aged non-Down syndrome patients
APP	amyloid precursor protein
Arg	arginine
Asn	asparagine
Asp	aspartate
AT	Republic of Austria
ATP	adenosine triphosphate
BDNF	brain-derived neurotrophic factor
CAT	catalase
CBS	cystathionine beta-synthase
CC	creative commons
CHDs	congenital heart defects
CIC	citrate carrier
Cit	citrulline
Cr	creatinine
Cu	copper (lat. *cuprum*)
Cys	cysteine
diTyr	dityrosine
DNA	deoxyribonucleic acid
DNs	dopaminergic neurons
DOI	digital object identifier
DS	Down syndrome
e.g.,	for example (lat. *exempli gratia*)
et al.	and others (lat. *et alia*)
etc.	and other similar things (lat. *et cetera*)
F	female
F_2_-dihomo-IsoPs	F_2_-dihomo-isoprostanes
F_2_-isoPs	F_2_-isoprostane
F_4_-NeuroPs	F_4_-neuroprostanes
FORD	free oxygen radical defence test
FORT	free oxygen radicals
FRAP	ferric reducing ability of plasma
FRM	free radical metabolism
fT4	free thyroxine
G6PDH	glucose-6-phosphate dehydrogenase
Gln	glutamine
Glu	glutamate
Glx	glyoxal
Gly	glycine
GPx	glutathione peroxidase
GPxs	glutathione peroxidases
GR	glutathione reductase
GSH	reduced glutathione
GS-Hb	glutathionyl-haemoglobin
GSHf	free glutathione
GSHt	total glutathione
GSSG	glutathione disulfide
GST	glutathione S-transferase
H_2_O_2_	hydrogen peroxide
HDL	high-density lipoprotein
His	histidine
HX	hypoxanthine
i.e.,	that is (lat. *id est*)
IE-NPBI	intraerythrocyte non-protein bound iron
IL-10	interleukin-10
IL-12	interleukin-12
IL-1α	interleukin-1 alpha
IL-2	interleukin-2
IL-6	interleukin-6
Ile	isoleucine
IMA	ischemia-modified albumin
iNOS	inducible nitric oxide synthase
IP	isoprostane
lat.	Latin
LDL	low-density lipoprotein
Leu	leucine
LMWA	enzymatic low molecular weight antioxidants
Lys	lysine
M	male
MCP1	monocyte chemoattractant protein-1
MDA	malondialdehyde
MDPI	Multidisciplinary Digital Publishing Institute
MEDLINE	Medical Literature Analysis and Retrieval System Online
Met	methionine
MGlx	methylglyoxal
MHR	methemoglobin reductase
mRNA	messenger ribonucleic acid
n	sample size
N	total sample size
N.S.	not significant
N/A	not applicable
NADPH	reduced nicotinamide adenine dinucleotide phosphate
NGF	tumour necrosis factor
NO	nitric oxide
non-DS	non-Down syndrome
NOS	nitric oxide synthase
O_2_^•−^	superoxide anion
OMIM	Online Mendelian Inheritance in Man
ONOO^−^	peroxynitrite
Orn	ornithine
OSDP	DNA/RNA oxidative stress damage products
PCO	protein carbonylation
PCs	protein carbonyls
Phe	phenylalanine
P-NPBI	plasma nonprotein-bound iron
PRISMA 2020	Preferred Reporting Items for Systematic Reviews and Meta-analyses
PROSPERO	International Systematic Review Registry
*p*-value	probability value
R	ratio
RNA	ribonucleic acid
RNS	reactive nitrogen species
ROS	reactive oxygen species
SA	sialic acid
SAH	S-adenosyl homocysteine
SAM	S-adenosylhomocysteine
Ser	serine
SOD	superoxide dismutase
SOD1	copper-zinc superoxide dismutase
SOD2	superoxide dismutase-2
STI	serum total iron
T3	triiodothyronine
TAOC	total antioxidant capacity of saliva
TAS	total antioxidant status
Tau	taurine
TBA	thiobarbituric acid
TBARS	thiobarbituric acid-reacting substances
TBG	thyroxine-binding globulin
TGF-β	transforming growth factor beta
TGs	triglycerides
th	ordinal indicator
tHcy	total homocysteine
Thr	threonine
TIBC	total iron binding capacity
TMR	transmembrane reductase
TNF-α	tumour necrosis factor alpha
tNO_x_	total nitrite and nitrate
TP	total protein
Trp	tryptophan
Trx	thioredoxin
Ts21	trisomy 21
TSH	thyroid stimulating hormone
tT4	total thyroxine
Tyr	tyrosine
UA	uric acid
UK	United Kingdom of Great Britain and Northern Ireland
Val	valine
X	xanthine
XO	xanthine oxidase
YDSP	younger Down syndrome patients
YnonDSP	younger non-Down syndrome
Zn	zinc (lat. *zincum*)
