# Supplementary material for: Oxidative Stress and Down Syndrome: A Systematic Review
Source: Antioxidants (Basel). 2025 Jul 2;14(7):816. doi: 10.3390/antiox14070816 (PMC12291806; doi:10.3390/antiox14070816)
Supplement: Supplementary file 1 [file antioxidants-14-00816-s001.zip › antioxidants-3582476-supplementary.pdf]

Supplementary Material

Supplementary File S1: Analysed Values from Body Fluids (Blood Except Serum and Plasma, Serum, Plasma, Saliva, Urine and Amniotic Fluid) in individuals with Down syndrome.

The following values were analysed from blood samples (except serum and plasma) in the included studies: serum total iron (STI), ferritin, transferrin, uric acid (UA), plasma nonprotein-bound iron (P-NPBI), intraerythrocyte non-protein bound iron (IE-NPBI), ROS, F_2-isoprostane (F_2-IsoPs), F_4-neuroprostanes (F_4-NeuroPs), F_2-dihomo-isoprostanes (F_2-dihomo-IsoPs), citrate, ATP-citrate lyase (ACLY), citrate carrier (CIC), 8-hydroxy-2′-deoxyguanosine (8-OHdG), malondialdehyde (MDA), glyoxal (Glx), glutathione disulfide (GSSG), nitric oxide (NO), lipid peroxidation, superoxide dismutase (SOD), catalase (CAT), glutathione peroxidase (GPx), adenosine deaminase (ADA), total antioxidant status (TAS), lipofuscin, glutathione S-transferases (GST), glutathione reductase (GR), total glutathione (GSHt), free glutathione (GSHf), hypoxanthine (HX), xanthine (X), allantoin (alla), methionine (Met), cystathionine, cysteine (Cys), xanthine oxidase (XO), vitamin E, vitamin C, thiobarbituric acid-reacting substances (TBARS), zinc (Zn), copper (Cu), free thyroxine (fT4), total thyroxine (tT4), thyroid stimulating hormone (TSH), triiodothyronine (T3), thyroxine-binding globulin (TBG) and thiobarbituric acid (TBA).

The following values were analysed from the serum sample: aspartate (Asp), glutamate (Glu), asparagine (Asn), serine (Ser), glutamine (Gln), histidine (His), glycine (Gly), threonine (Thr), citrulline (Cit), arginine (Arg), alanine (Ala), taurine (Tau), tyrosine (Tyr), valine (Val), methionine, tryptophan (Trp), phenylalanine (Phe), isoleucine (Ile), leucine (Leu), ornithine (Orn), lysine (Lys), uracil, beta-pseudouridine, uridine, hypoxanthine, xanthine, uric acid, sum of oxypurines, inosine, vitamin C, reduced glutathione (GSH), free oxygen radicals defense (FORD), nitrites, nitrates, lactate, creatinine (Cr), total cholesterol, high-density lipoprotein (HDL), low-density lipoprotein (LDL) and triglycerides (TGs).

The following values were analysed from the plasma sample: thiobarbituric acid, uric acid, vitamin C, vitamin E, reduced glutathione, glutathione disulfide, total glutathione, acid phosphatase (ACP1), methemoglobin reductase (MHR), transmembrane reductase (TMR), protein carbonyl, ferric reducing ability of plasma (FRAP), 4-hydroxynonenal (4-HNE), malondialdehyde, 25-hydroxyvitamin D (25(OH)D), protein carbonylation (PCO), total antioxidant status and thiobarbituric acid-reacting substances.

Total protein (TP), glutathione peroxidase, superoxide dismutase, total antioxidant capacity of saliva (TAOC), malondialdehyde, carbonylated proteins, uric acid, vitamin C, peroxidase, total antioxidant status, nitric oxide, sialic acid (SA), 8-hydroxy-2′-deoxyguanosine were analysed from the saliva sample.

From the urine sample, the following values were analysed: creatinine, total antioxidant status, 8-hydroxy-2′-deoxyguanosine, uric acid, thiobarbituric acid-reacting substances, allantoin, 2,3-dinor-8-iso-prostaglandin $F_{2\alpha}$-III (2,3-dinor-iP$F_{2\alpha}$-III), isoprostane (15-F_{2t}-IsoP), advanced glycation end products (AGEs), dityrosine (diTyr), hydrogen peroxide (H_2O_2) and total nitrite and nitrate (tNO_x).

In studies conducted on pregnant women carrying a foetus diagnosed with trisomy 21, an amniotic fluid sample was taken and the following parameters were analysed by cause: catalase, malondialdehyde, superoxide dismutase, glutathione peroxidase, adenosine deaminase, xanthine oxidase, nitric oxide, nitric oxide synthase (NOS), interleukin-6 (IL-6), interleukin-10 (IL-10), 25-hydroxyvitamin D, asprosin, advanced glycation end products, ischemia-modified albumin (IMA), alpha-1-antitrypsin (A1AT), DNA/RNA oxidative stress damage products (OSDP), protein carbonylation, 4-hydroxynonenal, thioredoxin (Trx), total glutathione, glutathione disulfide and isoprostane.
